# Gender Determination Through Mandibular Features on Orthopantomograms: A Preliminary Study

**DOI:** 10.7759/cureus.63790

**Published:** 2024-07-03

**Authors:** Abirami Arthanari, Vignesh Ravindran, Karthikeyan Ramalingam, Lavanya Prathap, Shaan Raj

**Affiliations:** 1 Department of Forensic Odontology, Saveetha Dental College and Hospitals, Saveetha Institute of Medical and Technical Sciences, Saveetha University, Chennai, IND; 2 Department of Pediatric and Preventive Dentistry, Saveetha Dental College and Hospitals, Saveetha Institute of Medical and Technical Sciences, Saveetha University, Chennai, IND; 3 Department of Oral Pathology and Microbiology, Saveetha Dental College and Hospitals, Saveetha Institute of Medical and Technical Sciences, Saveetha University, Chennai, IND; 4 Department of Anatomy, Saveetha Dental College and Hospitals, Saveetha Institute of Medical and Technical Sciences, Saveetha University, Chennai, IND; 5 Department of Forensic Medicine and Toxicology, Saveetha Dental College and Hospitals, Saveetha Institute of Medical and Technical Sciences, Saveetha University, Chennai, IND

**Keywords:** forensic identification, forensic odontology, sex determination, mandibular parameters, gender analysis, maximum ramus breadth, minimum ramus breadth, projective ramus height, coronoid ramus height, condylar ramus height

## Abstract

Introduction

Determination of gender can be highly accurate with a complete adult skeleton, but in scenarios like mass disasters, only fragmented bones might be available. In such cases, identifying gender relies significantly on which parts of the skeleton are found. The mandible is a notably distinct bone in the skull and can be key in determining gender, especially when the entire skull is not available. The mandibular features provide clues that can help forensic experts determine the gender of an individual.

Aim of the study

This preliminary study aimed to determine the gender of an individual using mandibular parameters such as coronoid ramus height, condylar ramus height, projective ramus, minimum ramus breadth, and maximum ramus breadth. The objectives of this study were to (i) determine the gender of an individual using various mandibular parameters, (ii) evaluate the effectiveness of these mandibular parameters in distinguishing between male and female individuals, and (iii) establish a reliable method for gender identification based on the measured mandibular parameters.

Materials and methods

Since it was a preliminary study, the sample size calculation was done using G*Power software (Version 3.1.9.4; Heinrich-Heine-Universität Düsseldorf, Düsseldorf, Germany). The sample size was determined to guarantee a 95% statistical power at a significance level (alpha error probability) of 0.05. To ensure sufficient statistical power, a total of 100 samples were included, with a projected sample size of 92. A total of 100 samples, evenly split between 50 males and 50 females aged 20 to 30 years, were analyzed. Orthopantomograms (OPGs) showing pathologies, fractures, developmental disturbances of the mandible, and edentulous mandibles were excluded from the study. Statistical analysis was performed using SPSS for Windows, Version 16.0 (Released 2007; SPSS Inc., Chicago, IL, USA). Additionally, an accuracy test, analysis of variance (ANOVA), multiple regression, and discriminant analysis for gender were performed on individual data.

Results

In this study, five mandibular parameters were analyzed for gender such as coronoid ramus height, condylar ramus height, projective ramus, minimum ramus breadth, and maximum ramus breadth showed a positive correlation comparatively, and a novel formula was developed.

Conclusion

According to the present study, panoramic radiography can be considered a valuable tool in sex determination (with an accuracy of 90%), and all parameters of the mandible exhibited sexual dimorphism, showing they are reliable parameters with a total accuracy of 90%. However, coronoid ramus height, projective ramus, and maximum ramus breadth played a significant role in identifying gender in this particular study.

## Introduction

Human skulls are vital in forensic odontology for identifying victims, especially using the mandible for gender determination. In forensic odontology, male and female skeletal features exhibit a spectrum of morphological configurations and metric values. The study of human growth, development, and evolution is fundamentally and irreversibly tied to a true understanding of the nature of human sexual dimorphism and the numerous elements that influence its expression. An integral component of all skeletal investigations is the isolation, interpretation, and quantification of sexual symptoms. Furthermore, it is crucial from a phylogenetic perspective to differentiate sexual distinctions from evolutionary modifications in hominid remains. The selection of skeletal features that can serve as useful sex indicators must be based on three fundamental criteria: they must endure the rigors of skeletonization and fossilization, have a morphology that reflects anatomical and/or physiological sex differences, and ideally, have a characteristic that is recognizable over time and across paleospecies. The most accurate location to determine an adult's sex, with a 95% accuracy rate, is the entire pelvis; however, unbroken pelvic remains are rarely found in prehistoric burials or fossil sites. Therefore, it is important to be able to maximize sex determination from as many bones as feasible since one does not always have the preferred bone in excellent shape. One of the strongest bones in the body is the mandible, frequently one of the few surviving fossilized hominids [1,2].

The morphology and metrics of the skull and mandible, soft tissues, forensic odontology, and deoxyribonucleic acid (DNA) testing on teeth can all be used to determine the gender of an unknown person. The mandible is the most durable and mobile component of the skull, serving as its primary point of articulation. With regard to age, sex, and race, alterations in its morphological characteristics can be observed. It serves as the working platform for dental surgeons. In forensic science and anthropology, the sex determination of bones is a crucial component of study because it forms the basis for further interpretations and analyses. To determine the sex of a bone, morphological and metric approaches are typically applied. Metric analysis has the advantage that the results can be easily compared to those from other investigations [3].

In everyday dental procedures, panoramic radiographs are frequently utilized to evaluate mandibular and maxillary important structures. They provide information about the majority of dental topics on a single film, making them a practical radiologic method for surveying dental problems. Panoramic radiographs are frequently prescribed, making them an effective tool for researching gender differences or correlations, as well as morphological changes that come with aging. Over the last 10 years, a plethora of studies have demonstrated the effectiveness of orthopantomograms (OPGs) in determining the morphological dimensions of the mandible [4].

The completeness of the remains and the level of sexual dimorphism present in the population are key factors in how accurately gender can be determined. Gender can be identified with up to 100% accuracy when the full adult skeleton is available for analysis, but in mass disaster situations where fragmented bones are typically discovered, sex determination depends heavily on the sections of the skeleton that are present [5]. The most frequently used part of the skeleton for gender identification is the skull, followed by the pelvis. The mandible, the most dimorphic bone in the skull, is crucial in determining sex in situations where the whole skull is absent. The largest and sturdiest bone in the face, the mandible, plays a significant role.

The mandible, which has a body that is horizontally curved and convex forward, is the largest and strongest bone in the face [6]. On the ramus are the coronoid and condyloid processes. Because it is the most durable bone in the facial skeleton and maintains its shape better than other bones, the mandible is seen as a promising subject for further study [7]. The relative difference in the musculoskeletal system's development, particularly the muscles of mastication linked to the mandible, may be the cause of sexual dimorphism in that region of the body. The word mandible comes from Latin and means "lower jaw." Mandere translates as "to chew." The term mandible is derived from this. The mandible is the face's largest, strongest, and lowest bone. Because the mandible keeps its shape better than other bones, it is used extensively in forensic osteology and anthropology [8].

In 2023, Girdhar et al. conducted a study that compared traditional lateral cephalometric tracing with modern digital analysis in 191 individuals (96 males, 95 females) aged 17 and older in India. Results indicated females generally had higher gonial angles (125.05° manual, 123.77° digital, 125.28° total) compared to males (122.583° manual, 121.715° digital, 122.008° total). Digital analysis achieved the highest gender estimation accuracy (60.7%), followed by Burstone’s method (57.1%) and manual tracing (56.5%). Burstone’s method identified more females correctly (57.9%), while digital (62.5%) and manual (59.4%) methods were better at identifying males. Despite digital methods showing higher accuracy, caution is advised against relying solely on one method for gender prediction, emphasizing the need for comprehensive approaches in forensic gender identification [9]. Odontology is commonly used in forensic contexts to identify human remains. Most studies on the gonial angle focused on lateral cephalograms, while fewer studies examined ramus height and bigonial breadth using both lateral cephalograms and OPGs for age determination from dental radiographs [10].

Sreelekha et al. conducted a study on 106 whole-adult human mandibles of unknown sex, aged between 18 and 60 years, collected at the Department of Anatomy and Forensic Medicine, Guntur Medical College, Guntur, India, from 2014 to 2015. They examined six parameters such as symphyseal height, mandibular body length, bicondylar diameter, bigonial diameter, inter-incisor width, and mandibular angle. Among these, the bigonial diameter and mandibular angle showed highly significant differences in sex determination, with accuracy rates of 82.15% and 81.5%, respectively. However, the study has several lacunae, especially since the sample size was relatively small and limited to a specific geographic location, potentially affecting the generalizability of the findings. Additionally, the study did not account for potential variations due to ethnicity, nutritional status, or other environmental factors that might influence mandibular characteristics. Furthermore, the accuracy rates, while significant, indicate that there is still a margin for error, suggesting a need for further research with larger and more diverse samples to validate and refine the findings [11].

So keeping this in mind, we conducted a preliminary study on 100 samples with equal sample size distribution in both genders using OPG to establish some parameters for gender determination and generated a novel formula for gender identification. The findings of this study may be useful in providing anthropological data that can be used in dental and medical practices as well. The main objectives of this study were to (i) determine the gender of an individual using various mandibular parameters, (ii) evaluate the effectiveness of these mandibular parameters in distinguishing between male and female individuals, and (iii) establish a reliable method for gender identification based on the measured mandibular parameters.

## Materials and methods

The current study was conducted within the Department of Forensic Odontology at Saveetha Dental College and Hospitals, Chennai, India, utilizing samples from the archive of the Department of Oral Medicine and Radiology. Using G*Power software (Version 3.1.9.4; Heinrich-Heine-Universität Düsseldorf, Düsseldorf, Germany), the sample size was determined to guarantee a 95% statistical power at a significance level (alpha error probability) of 0.05. To ensure sufficient statistical power, a total of 100 samples were included, with a projected sample size of 92. A total of 100 samples, evenly split between 50 males and 50 females aged 20 to 30 years, were analyzed. Mandibular OPGs of individuals aged 20-30 years are preferred in forensic research due to their stable developmental features, relevance to adult skeletal characteristics, availability of subjects for statistical analysis, adherence to legal and ethical standards, and practicality in conducting non-invasive examinations essential for detailed forensic investigations like gender determination, age estimation, and dental identification. The study included OPG radiographs with known sex, excellent clarity, and adequate contrast, excluding those with any developmental abnormalities, pathological conditions, or distortions. The project received approval from the Institutional Human Ethics Committee of Saveetha Dental College (IHEC/SDC/FACULTY/22/FO/059).

**Figure 1:**
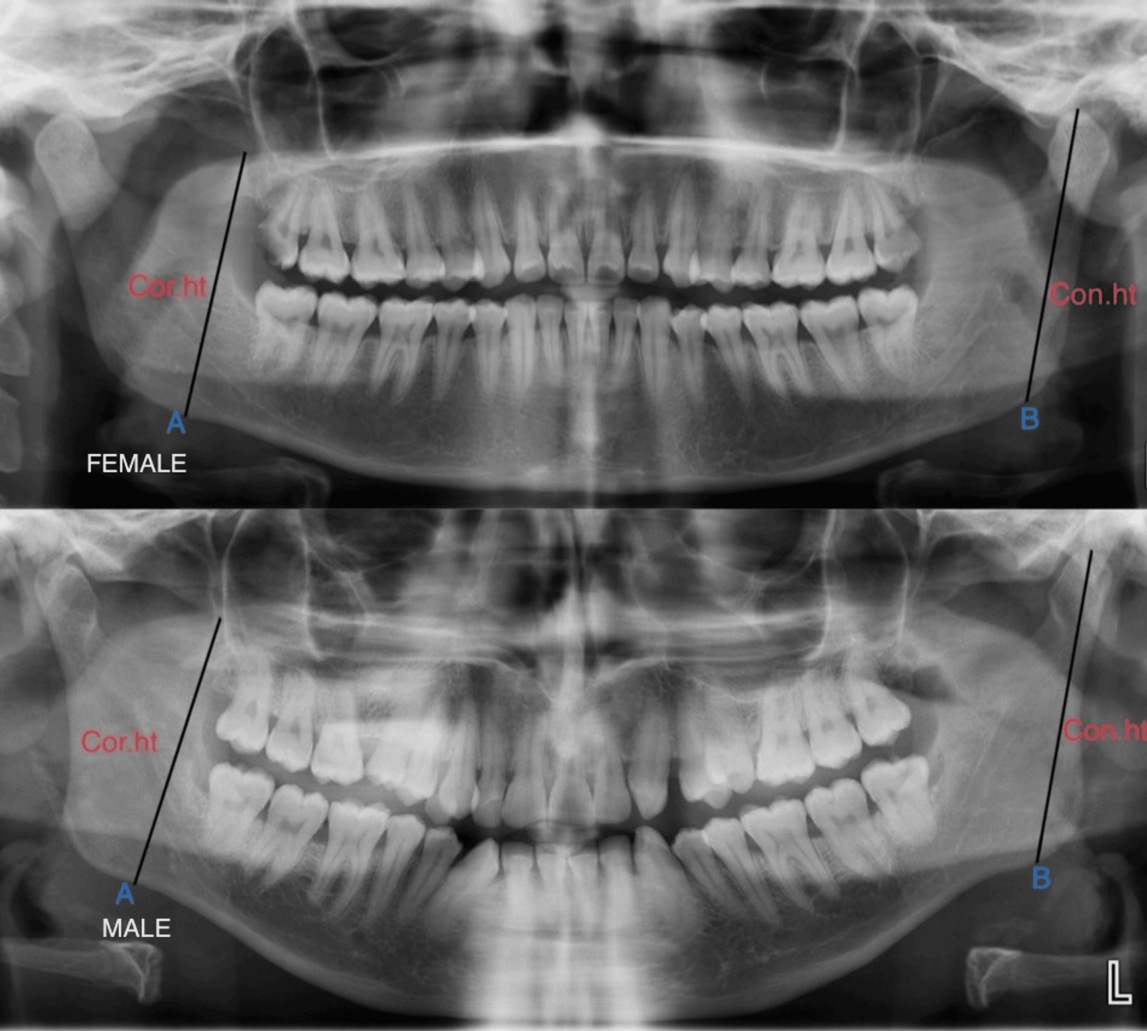
Representation of male and female orthopantomograms measuring various parameters like (A) condylar ramus height (Con. ht) and (B) coronoid ramus height (Cor. ht). Coronoid ramus height is measured from the projective distance between the coronoid and the most inferior point of the bone, and condylar ramus height is measured from the most superior point on the mandibular condyle to the most inferior point of the mandible.

Measurements of the samples were taken using Planmeca software (Version 6.0; Planmeca Romexis, Charlotte, NC, USA), and the results were entered into Excel (Microsoft® Corp., Redmond, WA, USA) for further statistical analysis. Digital OPGs had been obtained from Planmeca ProMax Scara 3 (70 kVp, 8 mA for 16 seconds; Planmeca Oy, Helsinki, Finland), with a 1:1 ratio. The digital OPG had been imported into Planmeca Romexis Viewer Software 2.9.2.R., and the measurements had been done. Microsoft Office Excel (2016) sheet was used for compiling the data. The statistical analysis was performed using SPSS for Windows, Version 16.0 (Released 2007; SPSS Inc., Chicago, IL, USA). In this study, an accuracy test, analysis of variance (ANOVA), multiple regression, and discriminant analysis for gender were performed on individual data. The parameters taken for the study included condylar height, coronoid height, projective ramus, maximum ramus breadth, and minimum ramus breadth. The representation of the coronoid ramus height of the mandible and the condylar ramus height of the mandible are shown in Figure 1.

**Figure 2:**
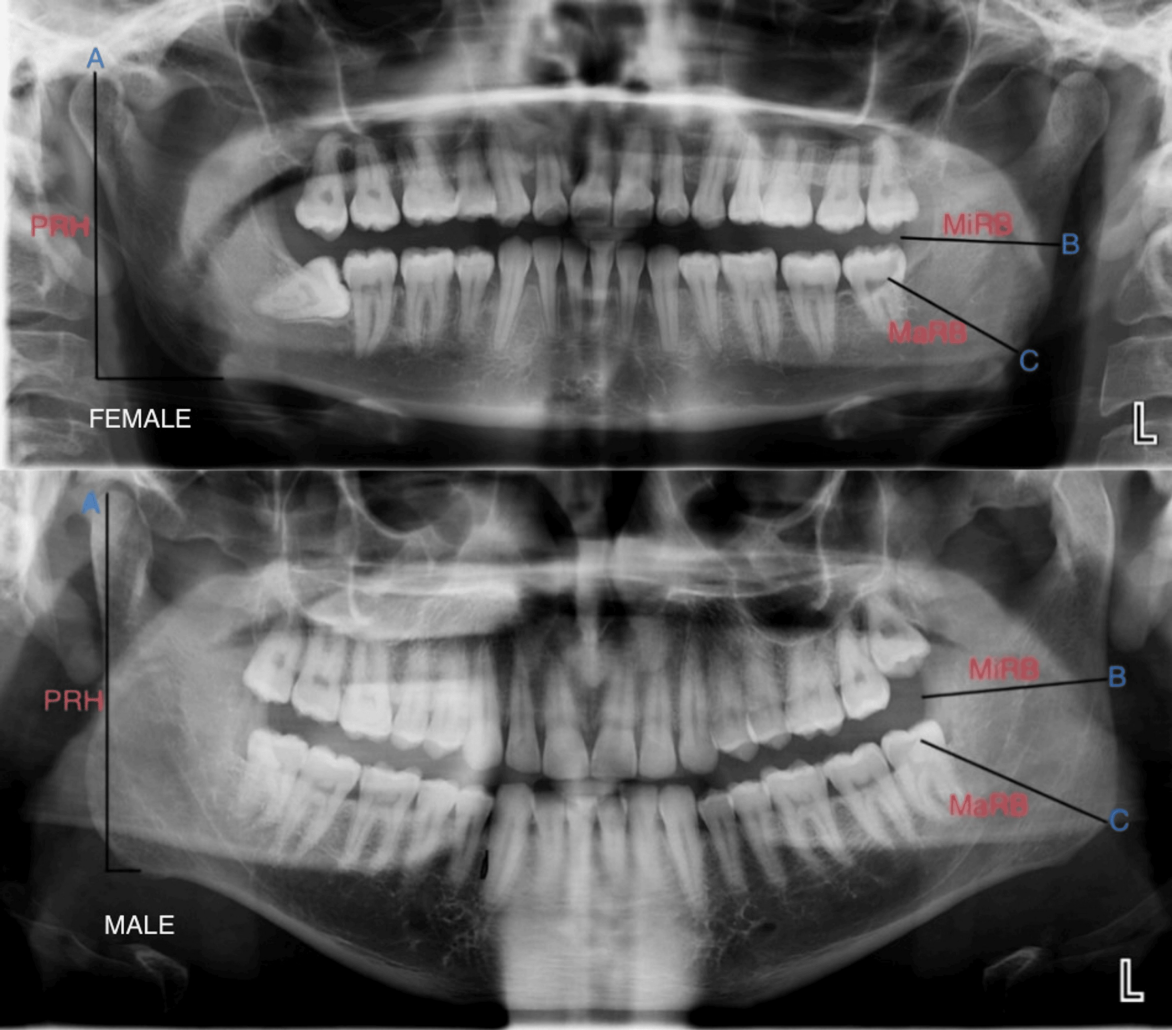
Representation of male and female orthopantomograms measuring various parameters like (A) projective ramus of the mandible (PRH), (B) minimum ramus breadth of the mandible (MRB), and (C) maximum ramus breadth (MaRB). The following parameters are projective ramus, which measures the distance between the highest point of the mandibular condyle and the lower margin of the bone; maximum ramus breadth, which measures the distance between the most anterior point on the mandibular ramus and a line connecting the most posterior point on the condyle and the angle of the jaw; and minimum ramus breadth, which measures the smallest anterior-posterior diameter of the ramus.

The representation of the projective ramus of the mandible, maximum ramus breadth, and minimum ramus breadth of the mandible are shown in Figure 2.

## Results

The results obtained in the present study have been segregated into five parameters, which were further divided into males and females. All statistical analyses were conducted for the p-value, and the data were examined using the Student's independent t-test. This present study was evaluated for the results of condylar height, coronoid height, projective ramus, maximum ramus breadth, and minimum ramus breadth. However, a few parameters were not adapted for sex analysis, and the null hypothesis was also not retained, as illustrated in Table 1.

**Table 1:** Descriptive statistics of all the mandibular parameters measured in the present study. Subjecting the values of the mandibular parameters to the analysis of variance, we find that the p-values of the coronoid height, projective ramus height, and maximum ramus breadth are 0.00*, while for the condylar ramus height it is 0.374, and for the minimum ramus breadth it is 0.050, i.e., less than 0.05, which makes them significant for sex determination. The Mann-Whitney U Test is performed to obtain the p-values of all mandibular parameters to check their probability. * p-values are less than 0.05, making the results statistically significant.

Mandibular parameters	Gender	Number (N)	Mean	Std. deviation	Std. error mean	Sig. p-value
Condylar height (mm)	Female	50	61.5440	11.88268	1.68046	0.374
	Male	50	60.9920	16.33257	2.30977	
Coronoid height (mm)	Female	50	64.3780	4.57987	0.64769	0.000*
	Male	50	69.0880	5.27275	0.74568	
Projective ramus (mm)	Female	50	67.1380	4.13244	0.58442	0.000*
	Male	50	78.0180	6.08922	0.86115	
Maximum ramus breadth (mm)	Female	50	40.8180	3.78163	0.53480	0.000*
	Male	50	46.4180	5.72159	0.80916	
Minimum ramus breadth (mm)	Female	50	35.8160	3.16714	0.44790	0.050
	Male	50	38.5680	4.83809	0.6821	

According to the population discriminant function, gender determination was analyzed, and a novel formula for analyzing gender was generated. The combined formula for gender analysis is given below for particular parameters, which are illustrated in Table 2.

**Table 2:** Discriminant analysis (stepwise regression method) for gender determination. The combined novel formula for gender analysis = 21.725+0.101*CH+0.164*PR+0.070*MRB and an individual formula for gender analysis is also given accordingly: (a) gender = 21.725+0.101*CH (coronoid ramus height); (b) gender = 21.725+0.164*PR (projective ramus height); (c) gender = 21.725+0.070*MRB (maximum ramus breadth).

Mandibular parameters	Canonical discriminant function coefficients
Constant	21.725
Coronoid ramus height (COR.HT)	0.101
Projective ramus (PRH)	0.164
Maximum ramus breadth (MARB)	0.070

In this present preliminary research, the classification of each gender was also segregated. Both female and male groups were analyzed for the prediction of gender according to the population. The results showed almost 90% accuracy in both male and female gender identification, as given in Table 3.

**Table 3:** Classification prediction of gender analysis. This table shows the results of a gender classification model, with 98% accuracy for predicting females and 82% for males. For females, 49 out of 50 were correctly predicted, while for males, 41 out of 50 were correctly predicted. The model is more accurate for females, suggesting potential bias or imbalance in the training data.

Gender		Predicted group membership		Total
		Female	Male	
Female		49	1	50
Male		9	41	50
Female (%)		98%	2%	100.0
Male (%)		18%	82%	100.0

## Discussion

Identification of human skeletal remains is a vital scientific endeavor in forensic medicine. Up to 100% accuracy in classifying a person's sex has been reported for the skull and pelvic bones; more recently, teeth have also been thought to be a helpful adjunct in this regard. The mandible bone is the most morphological, robust, and largest bone in the skull. It is very resistant to deterioration, making it a useful key in determining the sex of an individual. Furthermore, it has well-defined, standardized, and easily identifiable anatomical markers. Jaw length and width are regarded as the most significant cranial anthropometric measurements since they are utilized to calculate the cephalic index, which is a measure of cranial size [12]. Age and sex determination are crucial for identifying an individual, and these factors are interdependent. The age of an individual can be inferred based on their sex, and vice versa. Forensic odontologists play a significant role in determining age and sex using facial bones, particularly the mandible, which is known for its strength. The mandible exhibits sexual dimorphism and is a reliable bone for identification due to its dense compact structure. Its size, strength, and angulation are influenced by the muscles of mastication and the force exerted during mastication. The mandibular condyle and ramus, in particular, are the most sexually dimorphic regions as they undergo significant morphological changes and remodeling during growth. Therefore, this study focuses on the mandibular ramus for sex and age estimation [13].

**Figure 3:**
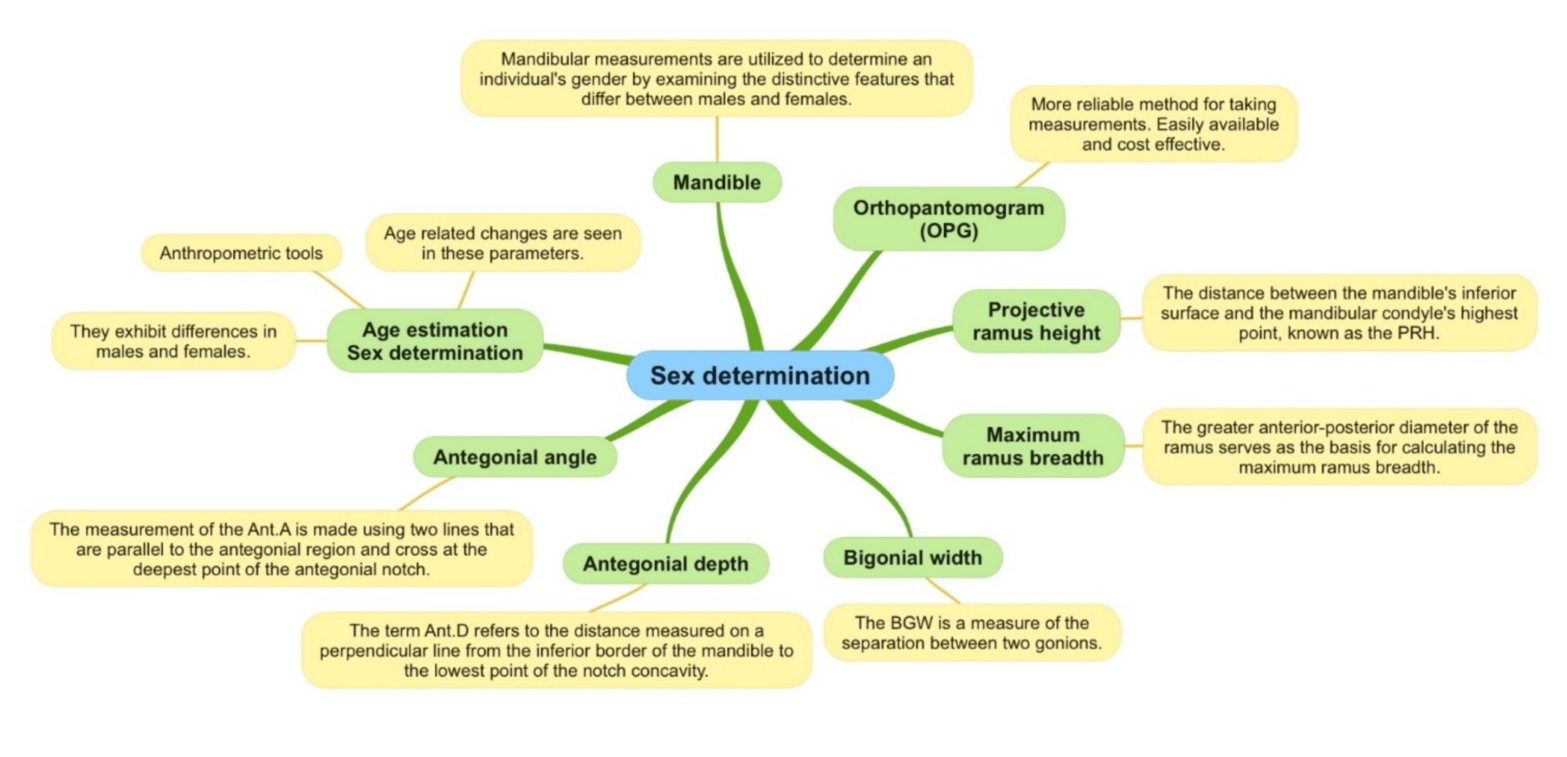
The figure explaining the mandibular parameters used in the study. PRH: Projective ramus height; BGW: Bigonial width; Ant. D: Antigonial depth; Ant. A: Antigonial angle

This study concentrated on various parameters of the mandible using OPGs. In particular, five variables were analyzed: coronoid ramus height, projective ramus, condylar ramus height, maximum ramus breadth, and minimum ramus breadth. Among these parameters, three (coronoid ramus height, projective ramus, and maximum ramus breadth) showed very good accuracy for gender identification, while the other two parameters were not applicable to gender identification in the Indian population. Classification prediction analysis also showed 90% accuracy in both female and male prediction of gender identification, and the representation of the mandibular parameters is given in Figure 3.

OPG analysis is a straightforward, noninvasive, cost-effective, and dependable approach for identifying the unidentified. OPGs are utilized as a screening tool for the diagnosis of oral illnesses, and they are also used to determine intraoral measures for comparative evaluation. Previous research has found that the coronoid process height has the highest potential for sex determination in the Indian population, with an accuracy of 71.45% when combined with other parameters such as the width of the coronoid process, the breadth of the mandibular ramus, the thickness of the mandibular structures, and its anatomical appearance. Various factors such as temporalis muscle attachment and action, unilateral chewing habit, and hormonal considerations cause variances in the morphology of the coronoid process [9,10]. 

Osteometric assessment using discriminant function analysis has been a widely employed method for estimating sex in unidentified skeletal remains. However, the levels of sexual dimorphism were population-specific due to a combination of genetic and environmental factors. Consequently, in various studies, discriminant function analysis has been conducted to identify the most significant parameter for sex estimation in the Egyptian population [14]. It was also found that the maximum breadth of the ramus of the mandible showed a significant difference between males and females, attributed to differences in bone remodeling between the sexes. However, the significant difference between males and females on both the right and left sides had not been observed in Group I (21-30 years) and Group II (31-40 years), possibly due to the smaller sample size [12].

Samantha et al. found similar findings to the current study, wherein the condylar height had been determined to be significant, with mean values for males and females of 65.34 and 61.69 mm, respectively, while the coronoid height had played no major impact. According to research, men had stronger muscles, which caused their mandibles to move faster during the chewing cycle. Consequently, the movement speed had increased [14].

Manoj et al. conducted a study using 500 panoramic view images, where visual tracing facilitated the assessment of both the left and right sides. They examined sexual dimorphism in the mandibular ramus, focusing on the shape of the sigmoid notch bordered by the coronoid and condylar processes. The shapes of the coronoid process, condyle, and sigmoid notch were interpreted. Results indicated that the most common sigmoid notch shape was the wide variant (41.2%), convex type for the condyle (50%), and round for the coronoid (53.4%). Asymmetry between the right and left sides was observed for the condyle (p-value = 0.001) and coronoid (p-value = 0.019). The study highlighted a higher occurrence of triangular-shaped coronoids in females (p-value = 0.012). The study concludes that sexual dimorphism in mandibular shape is influenced by factors such as masticatory activity, biting force, hormones, and genetics. These findings demonstrate that morphometric variations in the condyle, coronoid, and sigmoid notch can serve as valuable tools for personal identification [15].

Saini et al. utilized the mandibular ramus to demonstrate considerable sexual dimorphism, with an accuracy of 80.2%. A variation in the chosen sample size could have caused this inconsistent outcome. After puberty, sex could usually be determined with accuracy. An accurate approach to sexual dimorphism was sought by comparing the gonial and ramus dimensions in this early investigation on the pre-pubertal age group. In this investigation, it was discovered that the gonial angle was significant in females (p < 0.007), while the condylar, coronoid, and projection height of the ramus were significant in males (p < 0.001). For the ramus dimensions, the classification accuracy was 72%, and for the gonial dimensions, it was 66% [16].

Due to skeletal fragmentation, determining the gender of an unidentified skeleton in an explosion, conflict, or other mass calamity could be quite difficult. Skeletal remains identification was crucial for forensic medicine and anthropology, particularly for criminal investigations. The "Big Four" - sex, age, height, and ethnic background of the individual - were the primary indicators of biological identity in a forensic setting. Physicians, surgeons, medico-legal authorities, and anthropologists were able to correctly evaluate the findings of diagnostic operations on living people with the aid of the mandible and its changes in age, sex, and race [17].

Condyles have been a source of fascination for anthropologists from time immemorial, and it has been observed that men had longer condyles than women. Condyle size had often been measured in planes, such as anteroposterior and mediolateral, which could be more gender-specific. Different standard X-rays were used for condylar imaging, with panoramic views of each condyle provided by the OPG. The mandibular condyle is a developmental center with a unique shape as a functional joint unit. Age, gender, face type, intentional load, occlusal forces, and malocclusion type among the right and left facets have all been common variations within the condylar morphology. Including various parameters in one study has been useful to find out different values and has made it easier to determine gender in one population [18].

Limitations

Because of significant differences in mandibular development stages, growth rates, and longevity between sexes, the mandibular ramus can differentiate between them. A larger sample size must be used in future research. Furthermore, additional research in the adult age group is required to determine the accuracy of the formula derived from the current study, as growth spurts may influence the evaluation of gender differences.

## Conclusions

According to the present study, panoramic radiography can be considered a valuable tool in sex determination (with an accuracy of 90%), and all parameters of the mandible had sexual dimorphism, showing reliable parameters with a total accuracy of 90% in sexual dimorphism. The greatest and lowest breadths of the mandible's ramus, and the height of the coronoid, have not yet been studied and documented in the literature to determine a person's age in either a male or female. The current study found that, in comparison to males, females had lower values for the height of the coronoid indices. The mandible indices' maximum and lowest breadths had little bearing on identifying a person's sex. After analyzing the data, it was determined that coronoid ramus height, projective ramus, and maximum ramus breadth could be used to accurately determine the patient's age as well as sex. On the other hand, only older age groups and females can utilize the maximum and lowest breadth of the mandible's ramus to determine a person's sex. Given the small sample size in this study, further research with a larger proportion of samples could be required to definitively corroborate the findings.
